# Immunotherapy for Management of Thymic Epithelial Tumors: A Double-Edged Sword

**DOI:** 10.3390/cancers14092060

**Published:** 2022-04-20

**Authors:** Madison Ballman, Chen Zhao, Meredith J. McAdams, Arun Rajan

**Affiliations:** Thoracic and Gastrointestinal Malignancies Branch, Center for Cancer Research, National Cancer Institute, National Institutes of Health, Bethesda, MD 20892, USA; madison.ballman@nih.gov (M.B.); chen.zhao@nih.gov (C.Z.); meredith.mcadams@nih.gov (M.J.M.)

**Keywords:** thymoma, thymic carcinoma, immune tolerance, immunotherapy, immune-related adverse events, biomarker

## Abstract

**Simple Summary:**

Immunotherapy has a rapidly expanding role for the treatment of several cancers due to durable clinical activity and favorable tolerability. However, the unique biology of thymic epithelial tumors (TETs) increases the risk of immune-mediated toxicity. In this paper we review the biology of thymic cancers and its impact on the potential benefits and risks of immunotherapy. We describe the results of completed clinical trials of immune checkpoint inhibitors for advanced TETs and provide an overview of potential biomarkers of response or toxicity of immunotherapy that might influence future development of immunotherapeutic modalities for the treatment of advanced thymoma and thymic carcinoma.

**Abstract:**

Thymic epithelial tumors (TETs) are rare thoracic cancers that are broadly classified as thymomas and thymic carcinomas. Surgery is the cornerstone of management for early-stage disease. There are a limited number of effective treatment options for patients with advanced or recurrent disease. The occurrence of paraneoplastic autoimmune disorders in patients with TETs, especially thymomas, creates significant challenges for the development of immunotherapy, including immune checkpoint inhibitors, as a feasible treatment option. In addition, patients with TETs are at increased risk for the development of immune-mediated toxicity with a predilection for musculoskeletal and neuromuscular adverse events upon treatment with immunotherapy. The identification of biomarkers of response and toxicity is expected to play a key role in harnessing the benefits of immunotherapy for patients with TETs. In this paper we review the biology of TETs and the potential effects on the tolerability of immunotherapy. The results of clinical trials of immune checkpoint inhibitors for the treatment of advanced TETs are described to understand the potential risks and benefits of immunotherapy. We also provide an overview of future avenues for treatment with novel immunotherapeutic modalities and opportunities to develop biomarkers to improve the safety and tolerability of immunomodulatory treatments in patients with TETs.

## 1. Introduction

Thymic epithelial tumors (TETs) comprise a rare group of thoracic cancers, with an incidence of approximately 1.5 cases per million [1,2,3]. According to the World Health Organization’s histopathological classification, TETs are classified as thymomas, thymic carcinomas, and thymic neuroendocrine cancers [2,4,5]. Histologically, thymomas tend to resemble normal thymic architecture and contain a mixture of epithelial tumor cells and non-tumoral lymphocytes, in contrast to thymic carcinomas, which are epithelial cancers [2,5]. Thymomas are frequently associated with paraneoplastic autoimmune disorders (AD) due to underlying defects in immune tolerance [4]. The clinical outcomes in patients with TETs can exhibit significant variation and is partly influenced by histology [4]. While certain subtypes of thymomas tend to be relatively indolent, thymic carcinomas are aggressive tumors with high metastatic potential [4].

Surgical resection is the treatment of choice for early-stage disease and achievement of complete resection is one of the most important prognostic factors [2,3,4]. Advanced or metastatic disease is managed primarily with platinum-based chemotherapy [1,3]. Unfortunately, treatment options are limited for patients with relapsed or refractory disease [1]. A lack of actionable genomic alterations in TETs has created significant obstacles in the development of targeted therapies [6,7,8,9,10,11,12]. Hence, there is a pressing need to develop newer treatments for the management of patients with advanced TETs.

### 1.1. Thymus Physiology and Pathophysiology

The thymus plays a crucial role in lymphocyte development [3] and in the establishment of immune tolerance [1,13]. As lymphocyte progenitors move through the thymus, they undergo several modifications and eventually differentiate into mature lymphocytes [4,13]. These processes include both positive and negative selection [4]. Positive selection occurs in the cortex of the thymus and involves the preservation of double-positive T-cells which possess a T-cell receptor (TCR) that is capable of binding to cortical epithelial cells expressing the major histocompatibility complex (MHC) and self-peptides [1,3,4]. The positively selected double-positive T-cells then migrate to the medulla of the thymus for negative selection [4]. There, medullary thymic epithelial cells (mTECs) express tissue-specific self-antigens (TSAs); this expression is controlled by the transcription factors AIRE and FEZF2 [1,4,13,14]. mTECs with normal AIRE expression undergo rapid turnover leading to apoptosis, which causes them to release the TSAs to the dendritic cells [3,4,13]. The dendritic cells then present the TSAs to the developing T-cells [1,13]. Those that react against the TSAs too strongly are considered autoreactive and undergo apoptosis; this process constitutes negative selection [1,3,4,13]. Negative selection is crucial for the development of immune tolerance as it allows the immune system to recognize self-tissue and develop tolerance toward autoimmunity [1,13,14].

In TETs, however, immune tolerance is rendered dysfunctional due to the decreased expression of AIRE, FEZF2, and MHC class II, as well as altered thymic architecture [1,3,13]. Negative selection may be impaired by altered thymic architecture, which allows progenitor cells to evade the medulla where selection occurs, or due to issues with antigen presentation that are related to decreased expression of AIRE and FEZF2 in mTECs [2,3,13]. Furthermore, the decreased expression of these transcription factors can cause defective positive selection of immunosuppressive regulatory T-cells (Tregs) [13]. These changes, particularly those that affect negative selection, allow for the release of autoreactive T-cells which, in turn, predispose patients to autoimmunity [1,13].

However, these mechanisms alone cannot explain autoimmunity in TETs [2,3,13]. For example, AIRE expression is not typically affected in patients with B1 thymomas, yet they still experience high rates of autoimmune disorders (AD) [2]. Moreover, even patients with low or deficient AIRE expression in their tumor cells still possess preserved expression in their non-neoplastic thymic tissue [3]. Patients with thymoma also tend to have different symptoms and autoantibody profiles than individuals who have autoimmune disease solely due to absent AIRE expression [3]. Therefore, additional mechanisms likely contribute to the high rates of AD in patients with TETs [2]. One potential mechanism is the production of chemokines by the tumor which could induce metabolic derangements or cross-reactions between tumor neoantigens and tissue-related antigens, leading to the production of autoantibodies [2]. An improvement in many thymoma-associated paraneoplastic diseases with successful tumor-directed therapy lends support to this hypothesis [2,15]. Another potential factor contributing to autoimmunity is the presence of structural similarities between antigens that are overexpressed by tumor cells and autoantigens that are expressed on target organs [16]. For example, thymomas that are associated with myasthenia gravis (MG) overexpress the mid-sized neurofilament gene (NEF), which shares sequences coding for acetylcholine receptors and titin epitopes that are associated with MG [16]. Regardless of the mechanisms behind the high rates of paraneoplastic autoimmunity in patients with TETs, it is a key feature of the disease that must be considered when evaluating treatment modalities.

### 1.2. TETs and Autoimmunity

The clinical manifestation of a predisposition towards autoimmunity in patients with TETs, particularly thymomas, is the frequent occurrence of paraneoplastic AD [3,13,15]. The most common AD is myasthenia gravis, which occurs in 30–50% of patients with thymoma [3,13,15]. Other AD, such as systemic lupus erythematosus, pure red cell aplasia, polymyositis, and Good syndrome, are also associated with thymoma but are less common [3,13,15]. Paraneoplastic autoimmunity can be a presenting symptom of a TET or can develop several years after diagnosis and can occur even after thymectomy [3].

In a subset of patients with thymoma, paraneoplastic autoimmunity can also manifest clinically in the form of an immunodeficiency disorder due to the presence of anti-cytokine autoantibodies [17]. Clinically, these individuals appear to have an increased risk of developing opportunistic infections, including recurrent sinopulmonary infections, chronic mucocutaneous candidiasis, and disseminated varicella zoster [17].

## 2. Immunotherapy

**Figure 1:**
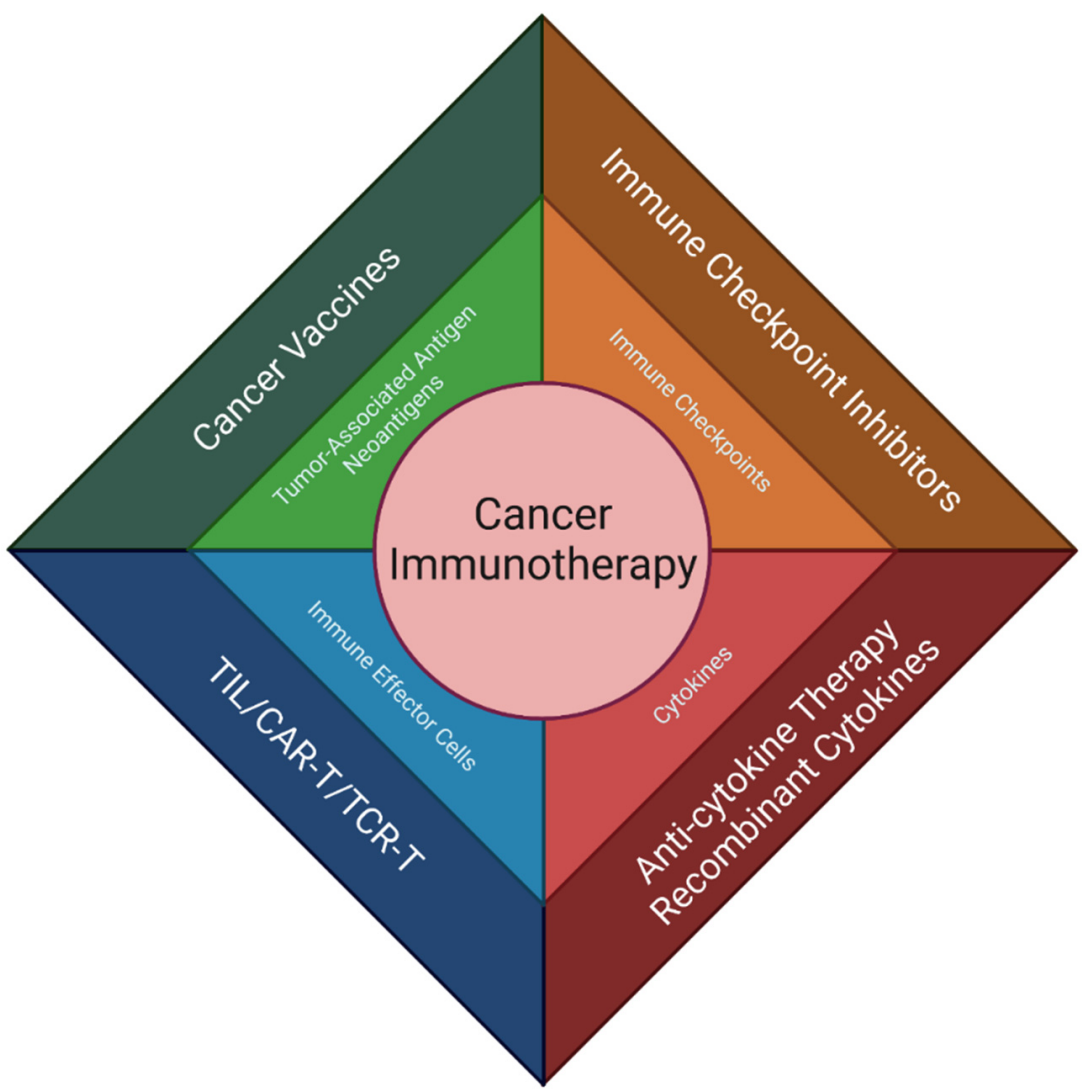
An overview of various forms of anti-cancer immunotherapy. The outer boxes outline different immunotherapeutic interventions. The inner boxes list corresponding immune targets.

Rapid advances in immunotherapy have revolutionized the management of several cancers. Immunotherapies are designed to activate an anti-tumor immune response with the goal of inducing meaningful and durable clinical responses against a variety of malignancies [18,19]. Anti-tumor immunity can be enhanced by immune checkpoint inhibitors (ICIs), cancer vaccines, cytokine-directed therapies, and adoptive cell therapies (Figure 1). ICIs are a class of drugs that enhance antitumor immunity by either blocking the signaling of inhibitory immune checkpoints or by enhancing the activity of immune stimulatory checkpoints, yielding augmented T-cell reactivity toward cancer cells [19,20]. Cancer vaccines are designed to stimulate a T-cell response towards tumor-associated antigens (TAAs) or neoantigens [18,20]. Cytokines, such as interferon alfa (IFNa), granulocyte-macrophage colony-stimulating factor (GM-CSF), interleukin (IL)-2, IL-12, IL-15, and IL-21, are immune messengers that can be used amplify a patient’s antitumor immune response [21]. Adoptive cell therapy (ACT) involves harvesting tumor infiltrating lymphocytes (TILs) from patients, manipulating their specificity and potency ex vivo, expanding their quantity, and administering them back into patients following lymphodepletion [18,20,22]. In doing so, the number of T-cells that are able to recognize TAAs, and thus kill the cancer cells, is increased [18]. ACT can also include the use of peripheral blood T-cells that are engineered to express a TCR targeting a specific tumor antigen through either co-culturing the T-cells with antigen-presenting cells that express that tumor antigen, or through genetic engineering [22]. Both of these ACT modalities have associated challenges, particularly the dependence of tumor antigen recognition on MHC presentation, which have led to the production of chimeric antigen receptor (CAR) T-cell therapies [18,19,22]. CARs are recombinant proteins that can be designed to recognize tumor antigens and are easily transfected into immune cells, leading to the rapid production of tumor-antigen specific T-cells [18,19]. Unlike other forms of ACT, CAR T-cell therapy allows for the recognition of antigens independent of MHC presentation [18,19,22]. These immunotherapies have transformed the treatment landscape for a myriad of cancers.

### 2.1. Immune Checkpoint Inhibitors

ICIs are engineered antibodies that are directed against negative immunologic regulators [23]. The regulators, or checkpoints, toward which ICIs are targeted, function to maintain immunologic tolerance by limiting T-cell activity [24]. Cancer cells can exploit these inhibitory pathways to escape immunosurveillance by expressing proteins which activate these inhibitory immune checkpoints [3]. ICIs function by inhibiting these negative checkpoints to enhance T-cell activity and generate an antitumor response [25]. Currently, FDA-approved ICIs target either the cytotoxic T-lymphocyte-associated antigen 4 (CTLA-4) or the programed death-1 (PD-1) pathways. CTLA-4 is upregulated after T-cell activation and downregulates T-cell function; it tends to act early in the process of T-cell activity [23]. PD-1, when engaged by its ligands PD-L1 or PD-L2, inhibits kinase signaling pathways that normally lead to T-cell activation, and primarily inhibits T-cell activity during the effector phase [23]. Since CTLA-4 and PD-1 affect different regulatory pathways, ICIs targeting these pathways have been developed and approved as monotherapies and combinatory therapies. Blocking these checkpoints generates clinical activity against a variety of malignancies [3,24], resulting in durable responses in a subset in patients [4]. The response rates for ICIs range from 15–30% in most solid tumors to 45–60% in patients with melanoma and MSI-H tumors [26].

Since ICIs are not solely directed at tumor-specific T-cells, their antitumor effects may be accompanied by the unintended activation of non-tumor-specific immune responses that target self-antigens, resulting in the development of immune-related adverse events (irAEs) [27]. These irAEs can affect any organ system within the body [27,28]. For CTLA-4-targeting ICIs, the most common irAEs are rash, pruritis, liver toxicity, diarrhea, colitis, and hypophysitis; for PD-1/PD-L1-targeting ICIs, the most common irAEs are cutaneous or gastrointestinal in nature [28]. Generally, CTLA-4 blockade is associated with more frequent and severe irAEs than PD-1/PD-L1-directed therapy, and the severity of irAEs tends to be exacerbated by combination therapy [27].

### 2.2. Justification for ICI Use in TETs

PD-L1 expression in tumor cells, high tumor mutational burden (TMB), and the presence of microsatellite instability are predictive of the response to ICIs [29]. TETs are PD-L1-expressing tumors, with expression frequencies of 23–92% in thymomas and 36–100% in thymic carcinomas [1], which provides a justification for the use of ICIs to treat TETs. Furthermore, emerging evidence suggests that PD-L1 expression might be even higher in clinically aggressive histological subtypes [30,31]. However, despite high levels of PD-L1 expression in TETs, its association with clinical outcomes is still unclear [32]. Indeed, the predictive value of PD-L1 as a biomarker differs across tumor types [33]. With regard to other validated biomarkers, TETs have the lowest TMB of all adult cancers [1,2,4] and microsatellite instability is uncommon [4]. Given the clinical activity of ICIs that are observed in patients with TETs, there are likely to be other, as of yet undiscovered, biomarkers of response to ICIs in patients with TETs. For example, a recent study examining ICI use in AIRE-deficient mice found that the antitumor effect of checkpoint inhibition was enhanced in mice with the deficiency, as opposed to wild-type mice [34]. This finding provides an additional justification for the use of ICIs in patients with TETs, as defective or deficient AIRE expression is commonly observed in association with thymomas [1,3,13].

### 2.3. Cancer Vaccines and TETs

The identification of TAAs whose epitopes are recognized by HLA class I-restricted cytotoxic T lymphocytes (CTLs) has spurred the devolvement of cancer vaccines [35]. One such TAA is transcribed by the Wilms’ tumor gene 1 (*WT1*). WT1 is overexpressed in various solid tumors, including lung, breast, thyroid, and colorectal cancers [35]. In a study of 13 thymic carcinoma and 5 thymoma tumor samples from advanced, pre-treated TETs, the overexpression of WT1 was detected in 84.6% of thymic carcinomas and 80% of thymomas [36]. The WT1 protein is responsible for many processes that promote oncological development, including cancer cell growth, resistance to apoptosis, cell migration, and tumor vascularization [36]. In non-TET malignancies, WT1 peptide vaccines have been shown to induce WT1-specific CTLs with anticancer activity [35]. The overexpression of WT1 in TETs provides an opportunity to develop cancer vaccines utilizing this TAA.

## 3. Clinical Evaluation of Immunotherapy for Treatment of Recurrent TETs

### 3.1. Clinical Activity of ICIs Targeting PD-1 or PD-L1 in TETs

Antibodies targeting PD-1/PD-L1 have been evaluated in four completed prospective trials (Table 1). Pembrolizumab, a PD-1 inhibitor, was evaluated in a Phase II trial in patients with advanced, refractory, or recurrent thymic carcinoma, and was associated with an objective response rate (ORR) of 23% [37]. Of these patients, 1 patient experienced a complete response, 8 patients experienced partial responses, and 21 patients (53%) experienced stable disease. With a median follow-up of 4.9 years (58.8 months), the median progression-free survival (PFS) was 4.2 months, the median overall survival (OS) was 2.1 years, and the median duration of response was 3.0 years [38]. The most common reason for treatment discontinuation was disease progression.

Another Phase II trial of pembrolizumab included patients with advanced thymoma and thymic carcinoma with disease progression after at least one line of platinum-based chemotherapy [32]. Treatment was associated with an ORR of 28.6% and 19.2%, and disease stabilization rates of 71.6% and 53.8%, for the thymoma and thymic carcinoma cohorts, respectively. After a median follow-up of 14.9 months both groups had a median PFS of 6.1 months. The median OS for the thymic carcinoma cohort was 14.5 months and was not reached for the thymoma cohort.

Avelumab, a PD-L1 inhibitor, has been evaluated in seven patients with recurrent thymoma and one patient with recurrent thymic carcinoma in a Phase I dose-escalation study [39]. An objective response was observed in four of the seven patients with thymoma and confirmed in two (29%) subjects. Avelumab is currently under investigation in a Phase II trial in patients with recurrent TETs (NCT03076554) [42]. Among 22 patients (12 thymoma; 10 thymic carcinoma) that were evaluable for response, the ORR was 17% and 20% for patients with thymoma and thymic carcinoma, respectively [40]. Stable disease was observed in 83% of patients with thymoma and 60% of patients with thymic carcinoma. After a median potential follow-up of 18.6 months, the median PFS for the thymoma and thymic carcinoma cohorts was 6.4 months and 14.7 months, respectively.

Nivolumab, a PD-1 inhibitor, has been evaluated in a Phase II trial (PRIMER study) of patients with recurrent thymic carcinoma [41]. While there were no objective responses, 73.3% of patients achieved disease stabilization. After a median follow-up of 14.1 months, the median PFS was 3.8 months, and the median OS was 14.1 months.

### 3.2. Safety and Tolerability of ICIs Targeting PD-1 and PD-L1 in TETs

ICIs appear to have a favorable safety profile in most patients with advanced thymic carcinoma. Common AEs that are associated with pembrolizumab include fatigue, anorexia, chest wall pain, cough, diarrhea, and transaminitis [32,37]. However, approximately 15% of patients receiving pembrolizumab can experience severe irAEs including myositis, myocarditis, myasthenia gravis, and hepatitis, which are usually responsive to treatment with high dose corticosteroids [32,37]. Similarly, nivolumab is generally well tolerated by most patients with thymic carcinoma. Common AEs include fatigue, fever, diarrhea, skin rash, electrolyte abnormalities, and transaminitis [41]. In the PRIMER study 13% of patients that were treated with nivolumab experienced serious irAEs including hepatitis and adrenal insufficiency [41].

In contrast, patients with thymoma that are treated with ICIs are at high risk for severe immune-mediated toxicity. A total of five (71.4%) out of seven patients with thymoma that were treated with pembrolizumab in a Phase II trial developed grade 3 or 4 irAEs, including myocarditis, hepatitis, thyroiditis, colitis, and nephritis [32]. Similarly, five (71.4%) out of seven patients with thymoma that were treated with avelumab in a Phase I trial developed irAEs including myositis, myocarditis, cranial neuropathy, and enteritis [39]. Treatment with high-dose corticosteroids with or without other immunosuppressive drugs resulted in complete resolution of irAEs in nearly 90% of patients with thymoma [32,39].

Taken together, these studies indicate that patients with TETs, especially thymomas, that are treated with ICIs are at higher risk for the development of potentially severe immune-mediated toxicity compared to patients with other malignancies. Moreover, multiple irAEs can occur concurrently, a phenomenon that is not observed often in individuals with other cancers that are treated with ICIs. Patients with TETs, irrespective of histology, also appear to have a poorly understood predilection for the development of muscle-related or neuromuscular autoimmune toxicity [32,37,39]. These observations support close follow-up and monitoring of patients with thymic carcinoma being considered for treatment with ICIs and avoiding use of ICIs for the treatment of thymoma except as a part of ongoing clinical trials.

Immune-mediated toxicity that was observed in patients with TETs that were treated with ICIs is summarized in Table 2.

### 3.3. Other Immunotherapeutic Interventions

In addition to ICIs, cancer vaccines have been prospectively evaluated for the treatment of advanced TETs. Oji and colleagues conducted a Phase II clinical trial examining a WT1 peptide vaccine in patients with advanced thymoma and thymic carcinoma [36]. Although no objective responses were observed, treatment was associated with disease stabilization in 75% of patients. The median time of the treatment was 683 days for the patients with thymoma and 133 days for the patients with thymic carcinoma. Adverse events following WT1 vaccination were infrequent, with the exception of grade 1 erythema and swelling, which was experienced by all the patients. There were two patients with thymoma that developed autoimmune complications, including pure red cell aplasia and myasthenia gravis after more than two years of treatment.

**Figure 2:**
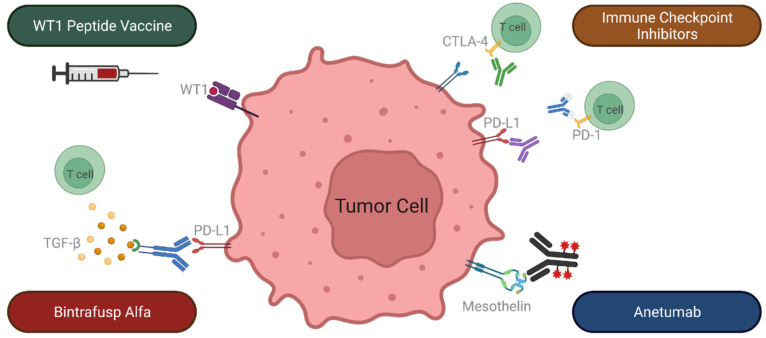
Immunotherapeutic interventions that are under investigation for treatment of recurrent thymic epithelial tumors. CTLA-4: cytotoxic T lymphocyte-associated antigen 4; PD-1: programed death-1; PD-L1: programed death-ligand 1; TGF-β: Transforming growth factor β.

Figure 2 illustrates some of the immunotherapeutic interventions under investigation for treatment of recurrent TETs.

### 3.4. Managing Immune-Mediated Adverse Events

The management of irAEs is based on the type and severity of toxicity [25,43,44]. The Common Terminology Criteria for Adverse Events (CTCAE) grading system provides an objective framework for the assessment of irAEs. Grade 1 irAEs generally do not require interruption of treatment unless these involve cardiac or nervous system toxicities, in which case all grades of toxicity require holding treatment for further workup and intervention [43,44]. Grade 2–4 irAEs require interruption of treatment, and possible discontinuation of immunotherapy for higher grades of toxicity [43,44]. All irAEs require comprehensive diagnostic workup, which should include an evaluation for alternative etiologies. The mainstay of treatment for irAEs is high-dose corticosteroids, which are given at dosages of 0.5 mg/kg to 2 mg/kg per day, depending on the type and severity of the irAE [25,43,44]. Patients should be informed about the potential side effects of steroid therapy and should receive prophylaxis against opportunistic infections if supra-physiologic doses (>10 mg of prednisone per day, or equivalent) are required for an extended period of time. The steroid dose is usually tapered gradually over several weeks with close monitoring of the irAE [25,43]. Although irAEs in patients with TETs are generally responsive to corticosteroids, additional interventions might also be required for the management of specific irAEs, such as plasmapheresis, intravenous immunoglobulins, rituximab, mycophenolate mofetil, azathioprine, and cyclophosphamide in case of a suboptimal response to steroids or for patients experiencing potentially life-threatening irAEs [25,43,44].

The treatment of irAEs with steroids and other immunosuppressive drugs raises concerns about the potential of these interventions to blunt the anti-tumor effect of immunotherapy [23]. Although prospective studies to evaluate the effect of these drugs on the efficacy of ICIs are lacking, limited retrospective data suggest that treatment of irAEs with immunosuppressive drugs does not have a substantial impact on efficacy endpoints such as ORR [45], OS, or time to treatment failure [46]. However, corticosteroid use at baseline appears to be associated with a decreased responsiveness to ICIs and shorter survival [47].

### 3.5. Re-Introduction of Immunotherapy after Treatment of irAEs

The decision to re-challenge patients who have previously experienced clinically significant irAEs with immunotherapy is challenging and involves a careful assessment of the risk and potential benefit [48]. Several studies have examined the impact of re-administration of the same ICI or a different class of ICI on anti-tumor activity and tolerability after resolution of serious irAEs [48,49,50,51,52,53]. Since anti-CTLA-4 and PD-(L)1 inhibitors utilize different immunologic pathways, the tolerability of switching ICI classes after experiencing severe irAEs with a particular type of ICI has been evaluated. One study examined the tolerability of PD-1-directed immune checkpoint inhibition in patients who had experienced ipilimumab (anti-CTLA-4)-associated immune toxicity [49]. While 37% of patients experienced irAEs after the re-challenge, most of these were new, rather than recurrent, irAEs. Additionally, the incidence of grade 3 or 4 irAEs was 21%, and the discontinuation of treatment was necessary in 12% of patients. These findings suggest that switching the class of ICI might be feasible for a subset of patients, particularly if the treatment options are limited. In addition to manageable toxicity upon re-administration of the same or a different class of ICI in patients who have experienced immune-mediated toxicity, some patients have also achieved an objective anti-tumor response which had not been observed following the initial treatment [50,54]. Studies specifically examining the resumption of PD-(L)1 therapy after prior treatment found that while roughly half of the patients experienced new or recurrent irAEs, the majority of these were mild and manageable [51,52,53]. Taken together, these data suggest that an ICI re-challenge may be considered in carefully selected patients after a discussion of the potential benefits and risks. However, this approach is not suitable for patients who have experienced severe or life-threatening immune toxicity and those who have required a prolonged course of immunosuppressive therapy for the management of irAEs.

The role of concurrent immunosuppression to decrease the risk of re-emergence of immune toxicity upon the resumption of treatment has also been examined in patients who developed irAEs following their initial treatment with an ICI. In one study, 14 patients who experienced severe ICI-related colitis were re-challenged upon the resolution of symptoms [51]. Of these, eight (57%) patients received vedolizumab (VDZ), an a4b7 integrin inhibitor, concurrently and experienced a substantially lower risk of recurrence of colitis compared with six patients who did not receive VDZ. Similarly, in another study five patients who had experienced gastrointestinal immune toxicity were re-challenged concurrently with TNF-a inhibitors and all patients tolerated the re-administration of immunotherapy without a recurrence of gastrointestinal symptoms [55]. In the context of TETs, preliminary observations from an ongoing Phase II trial of avelumab in recurrent TETs have demonstrated the ability to successfully re-administer avelumab with concurrent use of cyclosporine A for secondary prophylaxis in patients who had developed immune-mediated myositis [40]. These data are encouraging and support the need for further research to evaluate the safety and feasibility of the resumption of immunotherapy in patients with TETs who experience irAEs.

### 3.6. Biomarkers for Immunotherapy in the Context of TETs

#### 3.6.1. Biomarkers of Efficacy

PD-L1 expression and TMB are validated predictors of response to ICIs [29]. The correlation between PD-L1 expression and response of TETs to pembrolizumab has been evaluated in two independent Phase II trials [32,37]. High PD-L1 expression (positive staining in >50% of tumor cells) was associated with a greater likelihood of response and improved survival compared with low or absent PD-L1 expression [32,37]. Given these early findings, the role of PD-L1 expression as a predictive biomarker of response to ICIs in patients with TETs needs to be confirmed in future studies. Of note, published results are derived exclusively from patients with thymic carcinoma that are treated with a PD-1 inhibitor, and it remains to be determined if PD-L1 expression is a predictor of response and survival in patients that are treated with other ICIs and in individuals with thymoma and thymic neuroendocrine tumors.

The role of TMB as a biomarker of response to ICIs is somewhat debatable and of limited clinical relevance for patients with TETs that have a low TMB [4]. Hence, efforts are ongoing to identify novel biomarkers of response.

The evaluation of the genomic profile of patients with thymic carcinoma that are treated with pembrolizumab reveals the presence of *CYLD* mutations in responders and mutations of *BAP1* in non-responders [56]. Interestingly, these mutations appear to correlate with specific patterns of PD-L1 expression and it remains to be determined if responsiveness of thymic carcinoma to pembrolizumab is a function of the genomic characteristics of the tumor or the degree of PD-L1 expression.

An association between the clinical outcomes and the expression of interferon-g-related genes in patients with thymic carcinoma that were treated with pembrolizumab has yielded conflicting results leaving its role as a potential biomarker of response unclear [32,37,56].

In patients with thymoma that were treated with avelumab, responders were noted to have higher absolute lymphocyte counts, higher levels of TCR diversity, and lower frequencies of B-cells, regulatory T-cells, conventional dendritic cells, and natural killer cells before treatment compared with non-responders [39]. Further studies are necessary to validate these potential biomarkers of response since these observations are based on a small number of patients.

Finally, the development of irAEs in patients that were treated with ICIs has also been examined as a potential biomarker of response. An association between irAEs and an objective response has been observed with both pembrolizumab and avelumab in patients with recurrent thymic carcinoma and thymoma [32,39]. These observations are consistent with results from a comprehensive review of 30 studies evaluating the outcomes of treatment of various cancers with ICIs that found an association between the development of irAEs and longer survival [57]. This association was particularly significant for patients receiving PD-1 inhibitors who developed endocrine and dermatological irAEs. Despite these clinical observations, the biological mechanisms behind these relationships remain unclear and need to be explored in future studies.

Other novel biomarkers of response are under evaluation in patients that are treated with cancer vaccines. In patients that are treated with the WT1 peptide vaccine, WT1 delayed-type hypersensitivity (DTH) test positivity and the production of WT1-235 IgG antibodies have been evaluated as predictors of efficacy [36]. In a study examining the use of the WT1 peptide vaccine in patients with glioblastoma multiforme, both WT1-DTH positivity and the development of WT1-235 IgG antibodies were associated with longer survival [58]. Furthermore, a combination of a positive WT1-DTH test and WT1-235 IgG antibody production was a better predictor of PFS and OS than either test alone. In a trial of the WT1 peptide vaccine in patients with TETs, most individuals developed WT1-DTH positivity or WT1-235 IgG antibodies following vaccination, but few developed both [36]. As formal survival analysis was not conducted as part of this study, it remains to be determined if these changes are associated with an improvement in survival.

Ongoing efforts to discover additional biomarkers of response to immunotherapy are likely to improve the selection of patients with TETs for these treatments and improve clinical outcomes.

#### 3.6.2. Biomarkers of Safety

An increased incidence of irAEs among patients with TETs that were treated with ICIs has spurred the effort to identify biomarkers that predict the development of immune-mediated toxicity. Despite early evidence of an association between PD-L1 expression and anti-tumor response, there does not appear to be a relationship between PD-L1 expression and the development of irAEs [32,37].

However, in an analysis of peripheral blood mononuclear cells that were derived from patients with thymoma that were treated with avelumab, individuals who developed irAEs had B-cell cytopenia, lower levels of regulatory T-cells and conventional dendritic cells, and a higher degree of TCR diversity prior to the initiation of treatment [39]. Additionally, all individuals who developed treatment-associated myositis had detectable titers of acetylcholine receptor-binding antibodies and profound B-cell cytopenia at baseline [59]. Despite the small sample size, the use of these potential biomarkers of immune-related myositis can be considered for the appropriate selection of patients with TETs for immunotherapy.

## 4. Future Directions

### 4.1. Novel Immunotherapeutic Approaches for Treatment of TETs

Despite the role of ICIs in improving clinical outcomes, a minority of patients achieve an objective antitumor response and a subset of patients with TETs, especially thymomas, are not candidates for treatment due to presence of paraneoplastic AD. Hence, there is a pressing need for development of new therapeutic modalities for recurrent TETs and for the establishment of biomarkers that can identify patients that are most likely to benefit from treatment.

Several clinical trials are underway to evaluate combinations of ICIs with other anti-cancer therapies in order to improve responses, overcome de novo resistance, and modulate the tumor microenvironment.

Engagement of multiple immune checkpoints can be considered to activate non-redundant pathways [60]. The combination of CTLA-4 and PD-(L)1 inhibitors is approved for patients with metastatic melanoma, renal cell carcinoma, and subtypes of metastatic colorectal carcinoma [60], and a trial to evaluate the combination of nivolumab and ipilimumab in patients with thymic carcinoma and B3 thymoma is currently under way (NCT03134118) [61]. Ongoing trials are also assessing the combination of approved ICIs with drugs targeting other immune checkpoints, including TIM-3, TIGIT, LAG-3, and NKG2A receptors [60].

Another approach that is under evaluation is the combination of ICIs with other immunomodulating agents. Transforming growth factor β (TGF-β) is a cytokine that can contribute to tumor growth via tumor microenvironment modifications which promote invasiveness, migration, and metastasis [62]. Bintrafusp alfa, a bifunctional protein that functions as a TGF-β ‘trap’ and a PD-L1 inhibitor, is under evaluation in patients with previously treated advanced thymoma and thymic carcinoma (NCT04417660; Figure 2) [63]. Indoleamine 2,3-Dioxygenase 1 (IDO1) is an intracellular enzyme which depletes local tryptophan and increases the concentration of tryptophan metabolites [64]. This metabolic pathway results in effector T-cell apoptosis and the promotion of regulatory T-cells, generating an immunosuppressive tumor microenvironment [64]. Epacadostat, an IDO1 inhibitor, has been evaluated in combination with pembrolizumab in patients with advanced melanoma in a Phase III, randomized, clinical trial. Although the combination did not yield significant differences in PFS and OS compared to pembrolizumab monotherapy [64], it is also under evaluation in patients with thymic carcinoma (NCT02364076) [65]. Combinations of ICIs with traditional treatment modalities, such as chemotherapy, radiotherapy, and targeted therapies are also being studied in several clinical trials [60]. Examples of trials evaluating ICIs with anti-angiogenic therapies in patients with advanced TETs include the Phase II CAVEATT trial that consists of a combination of avelumab and axitinib in patients with advanced thymic carcinoma and B3 thymoma [66], and a Phase I/II trial of nivolumab and vorolanib in patients with thymic carcinoma (NCT03583086) [67]. Sunitinib, a multikinase inhibitor with antiangiogenic and immunomodulatory properties, has already demonstrated clinical activity in patients with thymic carcinoma [10] and is currently being investigated in conjunction with pembrolizumab for the same indication (NCT03463460) [68].

Attempts to develop novel immunotherapeutic combinations for patients with recurrent TETs represent important advances that aim to synergistically strengthen the antitumor effect of ICIs and produce more frequent and durable responses.

Another promising immunotherapeutic approach involves the targeting of novel cancer antigens. One such target that is under investigation is mesothelin, a cell-surface antigen that is normally found on mesothelial cells lining the pleura, peritoneum, and pericardium, and which is highly expressed in several types of cancer [69,70]. Strong cell surface expression of mesothelin is frequently observed in thymic carcinomas, but only infrequently in thymomas, and is absent in thymic neuroendocrine tumors [69]. Several different approaches for targeting mesothelin are under investigation, including the use of antibody-drug conjugates [70]. Anetumab ravtansine, an anti-mesothelin antibody conjugated to a tubulin inhibitor, is currently being evaluated in a variety of mesothelin-expressing tumors, including thymic carcinoma (NCT03102320; Figure 2) [71]. CAR T-cell therapy represents another modality for targeting mesothelin, by modifying autologous T-cells to express a mesothelin-binding T-cell receptor, such that binding of these cells to mesothelin activates an anti-tumor response [70]. While the use of mesothelin-directed CAR T-cell therapy has been explored in patients with solid tumors [72], its role in the treatment of thymic carcinomas is yet to be evaluated.

Another antigen that can be harnessed for the development of CAR T-cell therapy is CD70, a protein belonging to the tumor necrosis family [73,74], which mediates the interaction between B- and T-lymphocytes [73]. CD70 is an appealing immunotherapeutic target due to its low expression in non-neoplastic cells, including a limited subset of normal lymphocytes and dendritic cells [75], and high expression in many hematologic cancers and some solid tumors [73,74,75]. An in vitro investigation of CD70-targeting CAR T-cells found that the administration of the modified T-cells resulted in T-cell activation, CD27 co-stimulation, and recognition and killing of CD70-positive tumor cell lines and primary tumor samples [75]. Furthermore, an in vivo murine model showed that CD70-targeting CAR T-cells generated sustained antitumor activity [75]. These findings suggest that CD70-directed CAR T-cell therapy may be a promising option for the treatment of CD70-positive malignancies. Consequently, CD70-directed CAR T-cells, both in combination and alone, are currently being assessed in patients with advanced B-cell malignancies and other malignant hematological diseases (NCT03125577; NCT04662294) [76,77]. Since approximately 79–88% of thymic carcinomas express CD70 [73,74], the possibility of evaluating CD70-directed CAR T-cell therapy for advanced thymic carcinomas should be considered in the future.

The use of immunotherapy with or without chemotherapy and radiation therapy in the perioperative period for patients with early-stage or locally advanced disease is an area of active research. Ongoing clinical trials evaluating neoadjuvant immunotherapy in patients with stage I-III, resectable NSCLC, have shown that PD-1/PD-L1-targeting ICIs have excellent activity and tolerability compared with neoadjuvant chemotherapy, including higher rates of pathological complete response (pCR) [78], and are, therefore, likely to find a role in the treatment of early-stage NSCLC [79]. With reference to thymic cancers, a neoadjuvant immunotherapy/chemotherapy combination is currently under evaluation in patients with locally advanced TETs, to determine operability following treatment (NCT03858582) [80].

Table 3 provides an overview of ongoing clinical trials evaluating immunotherapy for patients with TETs.

### 4.2. Biomarker Development

Currently, there are few validated biomarkers that predict ICI efficacy. Furthermore, some validated biomarkers, such as PD-L1 expression, may be imprecise in their predictive value, with demonstrable clinical activity in individuals lacking biomarker expression [81]. To increase accuracy and predictive value, novel biomarkers of response are under investigation.

Transcriptome analysis provides an avenue to identify new biomarkers of response to immunotherapy [82]. For example, an immuno-predictive score (IMPRES), which encompasses 15 pairwise transcriptomics relationships between immune checkpoint genes, has been developed to identify individuals with melanoma that are likely to respond to ICIs [83]. Additional studies are necessary to determine if IMPRES will retain predictive value for other malignancies [82,83]. Similarly, TIDE is a computational method that models two mechanisms of tumor immune evasion—the induction of T-cell dysfunction and the prevention of T-cell infiltration, in order to predict response to ICIs [84]. TIDE was successful in predicting response to ICIs in patients with melanoma with greater accuracy than other biomarkers, such as PD-L1 expression and TMB [84]. The utility of TIDE for patients with other types of tumors remains to be determined [82,84].

In addition to generating gene expression signatures, transcriptome analysis can be used to identify specific biomarkers for response. Using biopsies from a pan-cancer, predominantly pre-treatment cohort, whole genome and transcriptome analysis (WGTA) has been used to develop CD8+ T-cell and macrophage expression scores that predict for improved survival [85]. While further studies are necessary to validate these immune-related biomarkers, consistency across a variety of cancer types makes these approaches promising [85]. The inclusion of novel biomarkers such as those that are described above in prospective clinical trials for patients with TETs should be considered to improve patient selection and identify patients that are most likely to benefit from ICIs.

Biomarkers to predict toxicity are of particular relevance for patients with TETs under consideration for immunotherapy due to a higher risk for developing immune-mediated toxicity.

Several potential biomarkers have been examined for an association with irAEs. For example, a study examining cytokines and chemokines in patients with melanoma that were treated with ipilimumab found that a higher baseline level of circulating IL-17 was associated with the development of severe immune-mediated colitis [86]. Conversely, lower levels of circulating IL-6 at baseline were associated with a higher likelihood of developing irAEs, particularly colitis, in patients with melanoma that were treated with ipilimumab [87,88]. Further investigation is necessary to evaluate the role of these cytokines as biomarkers of toxicity in patients with TETs.

Microbiome composition has also been examined as a predictor of ICI toxicity. Studies in patients with melanoma have demonstrated that baseline microbiota compositions with a higher representation of bacteria belonging to the *Bacteroidetes* phylum are associated with resistance to the development of CTLA-4-induced colitis [88,89]. Conversely, patients with baseline microbiota that are enriched with *Faecalibacterium* genus and other *Firmicutes* tend to have a higher incidence of colitis-related irAEs [88]. In patients with lung cancer that are treated with immunotherapy alone or chemoimmunotherapy, baseline enrichment of *Bifidobacterium* and *Desulfovibrio* in the gut microbiota was significantly associated with a lower incidence of treatment-related irAEs [90]. It will be intriguing to determine if there is a relationship between baseline microbiome compositions and the development of irAEs in individuals with TETs.

Finally, genomic analysis has been used to identify somatic mutations that are associated with irAEs. In a study involving whole exome sequencing analysis of 87 tumor samples from 49 patients with metastatic melanoma that were treated with PD-1 and CTLA-4 inhibitors, patients who developed colitis were found to have tumors that were enriched with 14 mutated genes compared with patients who did not develop colitis [91]. Additionally, patients who experienced any irAE were noted to have enrichment of seven mutated genes as compared to the patients who did not develop an irAE. These findings suggest that specific mutations within tumors may contribute to the development of irAEs and that mutational patterns can be considered for evaluation in patients with TETs that are receiving immunotherapy.

### 4.3. Consideration of Patients with Paraneoplastic AD for Immunotherapy

Individuals with AD are usually excluded from clinical trials evaluating ICIs due to concerns about triggering severe immune toxicity [25]. This exclusion is particularly restrictive for patients with thymoma since the disease is frequently associated with paraneoplastic autoimmunity [3]. Clinical trials examining ICIs in patients with melanoma and NSCLC with pre-existing AD have found that while irAEs occurred at an increased frequency compared with patients without AD, they were generally mild and could be managed with standard immunosuppressive treatments [92]. Similarly, AD flares that occurred were also manageable and infrequently led to treatment discontinuation [92]. Patients with active AD tended to have higher rates of autoimmune flares than those with inactive AD, indicating that it is safer for patients with minimally active or inactive AD to be considered for ICI therapy [49,93]. Importantly, patients with AD experienced similar response rates to those without, even though many individuals were concurrently receiving immunosuppressive or immunomodulatory drugs [92].

As with patients who have previously experienced irAEs, concurrent immunosuppression could improve the safety of ICI therapy in patients with advanced cancers and pre-existing AD without abrogating anti-tumor activity. Although some studies have found that baseline use of corticosteroids at a dose of >10 mg per day of prednisone (or equivalent) is associated with a decreased response to PD-(L)1-directed therapy and shorter survival [47], the association between these variables remains controversial, and it remains to be determined if concurrent use of corticosteroids can reduce the clinical activity of ICIs [94]. Interestingly, it appears that selective immunomodulatory agents may not affect ICI efficacy and may therefore be a plausible alternative to non-selective agents, such as corticosteroids, in the management of AD [95,96].

Prospective clinical trials are necessary to further examine the role of concurrent immunosuppressant usage in patients with pre-existing AD, and its effect on ICI efficacy. If feasible, this approach is likely to increase the prospects of considering immunotherapy for patients with TETs and a history of AD.

## 5. Conclusions

Immunotherapy is being increasingly evaluated as a treatment option for patients with recurrent TETs. A subset of patients with advanced disease derives durable clinical benefit from ICIs. Combinations of ICIs with other systemic therapies, including other forms of immunotherapy, are under evaluation to improve clinical outcomes. However, defects in immune self-tolerance that are associated with thymic cancers increase the risk for severe immune-mediated toxicity. Further research is needed to develop strategies to treat patients with thymoma and paraneoplastic AD with immunotherapy, and safely reintroduce immunotherapy for individuals deriving clinical benefit who have previously experienced immune-mediated toxicity. Ultimately, a greater understanding of thymic biology and the development of novel predictive biomarkers is required to make immunotherapy a safe and feasible option for patients with thymoma and thymic carcinoma.

## Figures and Tables

**Table 1:** Clinical activity of immune checkpoint inhibitors in patients with thymic epithelial tumors.

ICI Type	Number of Patients	Response Rate (%)	Disease Stabilization (%)	Median PFS (Months)	Median OS (Months)
Pembrolizumab [37,38]					
Thymic carcinoma	40	22.5	52.5	4.2	24.9
Pembrolizumab [32]					
Thymoma	7	28.6	71.4	6.1	Not reached
Thymic carcinoma	26	19.2	53.8	6.1	14.5
Avelumab [39]					
Thymoma	7	57.1	28.6	NR	NR
Thymic carcinoma	1	0	100	NR	NR
Avelumab [40]					
Thymoma	12	16.7	83.3	6.4	NR
Thymic carcinoma	10	20.0	60.0	14.7	NR
Nivolumab [41]					
Thymic carcinoma	15	0	73.3	3.8	14.1

ICI: Immune checkpoint inhibitor; PFS: Progression-free survival; OS: Overall survival; NR: Not reported.

**Table 2:** Immune-related adverse events that were experienced by patients with thymic epithelial tumors following immune checkpoint inhibition.

irAE N(%)	Pembrolizumab [37]	Pembrolizumab [32]		Avelumab [39]		Nivolumab [41]
	TC	TC	Tm	TC	Tm	TC
	N = 40	N = 26	N = 7	N = 1	N = 7	N = 15
Elevated AST				0	4 (57.1)	8 (53.3)
Elevated ALT				0	4 (57.1)	3 (20)
Hepatitis	4 (10)	2 (7.7)	2 (28.6)			
Transaminitis	1 (2.5)					
Colitis ^A^	0	0	1 (14.3)	0	0	3 (20)
Enteritis	0	0	0	0	1 (14.3)	0
Myasthenia gravis	1 (2.5)	2 (7.7)	1 (14.3)	0	0	0
Polymyositis	3 (7.5)	0	0	0		
Elevated CPK	3 (7.5)	0	0	0	4 (57.1)	3 (20)
Myocarditis	2 (5)	0	3 (42.9)	0	3 (42.9)	0
Subacute myoclonus	0	1 (3.8)	0	0	0	0
Cranial neuropathy	0	0	0	0	1 (14.3)	0
Thyroiditis ^B^	0	1 (3.8)	2 (28.6)	0	0	1 (6.7)
Pancreatitis	1 (2.5)	0	0	0	0	0
Diabetes mellitus type I	1 (2.5)	0	0	0	0	0
Nephritis ^C^	0	0	1 (14.3)	0	0	2 (13.3)
Adrenal Insufficiency	0	0	0	0	0	1 (6.7)
Dermatitis	0	0	2 (28.6)	0	0	0
Pruitis	0	3 (11.5)	0	0	0	0
Skin rash	0	2 (7.7)	0	0	0	4 (26.7)
Bullous pemphigoid	1 (2.5)	0	0	0	0	0
Conjunctivitis	0	0	1 (14.3)	0	0	0

^A^ Includes three cases of diarrhea that were classified as immune-related adverse events. ^B^ Includes one case of hypothyroidism that was classified as an immune-related adverse event. ^C^ Includes two cases of elevated creatinine that were classified as immune-related adverse events. irAE: immune-related adverse event; TC: thymic carcinoma; Tm: thymoma; AST: aspartate transaminase; ALT: alanine transaminase; CPK: creatine phosphokinase.

**Table 3:** Ongoing clinical trials of novel immunotherapeutic modalities in patients with thymic epithelial tumors.

Intervention	Modality	Target	Patient Population	Trial
Nivolumab, Ipilimumab	Combinatory Immunotherapy	PD-1, CTLA-4	Thymic carcinoma, B3 thymoma	NCT03134118 [61]
Bintrafusp alfa	Combinatory Immunotherapy	PD-L1, TGF-β	Thymic carcinoma, thymoma	NCT04417660 [63]
Pembrolizumab, Epacadostat	Combinatory Immunotherapy	PD-1, IDO1	Thymic carcinoma	NCT02364076 [65]
Avelumab, Axitinib	Immunotherapy + Targeted Therapy	PD-L1, VEGFR	Thymic carcinoma, B3 thymoma	2017-004048-38 [66]
Nivolumab, Vorolanib	Immunotherapy + Targeted Therapy	PD-1, VEGFR, PDGFR	Thymic carcinoma	NCT03583086 [67]
Pembrolizumab, Sunitinib malate	Immunotherapy + Targeted Therapy	PD-1, VEGFR, PDGFR, CSFR	Thymic carcinoma	NCT03463460 [68]
Anetumab ravtansine	Cancer Antigen Targeting Therapy	Mesothelin	Thymic carcinoma	NCT03102320 [71]
Pembrolizumab	Neoadjuvant Immunotherapy	PD-1	Thymic carcinoma, thymoma	NCT03858582 [80]

PD-1: programed death-1; CTLA-4: cytotoxic T lymphocyte-associated antigen 4; PD-L1: programed death-ligand 1; TGF-β: Transforming growth factor β; IDO1: Indoleamine 2,3-Dioxygenase 1; VEGFR: vascular endothelial growth factor receptor; PDGFR: platelet-derived growth factor receptor; CSFR: colony-stimulating factor receptor.
